# One person’s meat is another’s poison: representations of the meat-health nexus in UK news media

**DOI:** 10.1093/heapro/daac072

**Published:** 2022-07-05

**Authors:** Gilly Mroz, James Painter

**Affiliations:** Department of Zoology, University of Oxford, Oxford, UK; Reuters Institute for the Study of Journalism, University of Oxford, Oxford, UK; School of Geography, University of Oxford, Oxford, UK

**Keywords:** meat, health and nutrition, UK media, content analysis

## Abstract

Mainstream media play a central role in shaping the ways diet and nutrition are discussed in the public sphere, yet few studies have explored its depictions of the meat-health nexus. Focusing on eight of the most popular news online sites consumed by lower-income groups in the UK—the demographic most likely to eat meat, according to a survey conducted for this study—we carried out content analysis of 128 articles. We found, first, a multiplicity of pro- and anti-meat narratives across all news outlets; second, that the dominant recommendation, found in 40% of our sample, was to eat less or no red meat; and third, that a balanced or neutral sentiment was present in over half of our sample, with a ratio of 3:2 (anti-versus pro-meat) in remaining articles. We found that the editorial leaning of a news outlet was not closely correlated with its overall sentiment towards meat consumption; all were neutral or slightly anti-meat, with the exception of LAD Bible, the only clearly pro-meat outlet. Qualitative analysis uncovered three key themes: the risk of red meat on colorectal cancer, uncertainty around plant-based options, and individual dietary choice. We use case studies guided by these themes to highlight some of the shortcomings of health communication and provide recommendations, with a focus on improved dialogue between journalists and researchers.

## Introduction

An increasing number of people in western countries are choosing to follow more plant-based diets, in which animal products are limited or excluded and plant-derived foods are emphasised. According to a 2021 YouGov survey commissioned for this study, 9% of adults in the UK now describe themselves as vegan or vegetarian. Participation in Veganuary—an annual campaign to eat only vegan foods throughout the month of January—is also increasingly popular: participants come from over 200 countries and territories, and a record number (582,538) reportedly signed up to the campaign in 2021 (Veganuary, 2021). It is now easier than ever to find plant-based options, with all the UK supermarkets having introduced their own vegan ranges (Butler, 2018) and fast-food outlets serving plant-based alternatives to their meat-based bestsellers (Walker, 2020). The trend is reflected in recent market figures: in 2020 the UK was the largest European market for plant-based meat alternatives and the second largest for plant-based milks (Smart Protein, 2021).

One of the most common reasons for western consumers to reduce or eliminate meat from their diet is the perceived health benefits of plant-based eating, surpassing animal welfare and environmental concerns (Mullee et al., 2017; Jones, 2020). A growing body of research suggests that high-level meat consumption is associated with non-communicable diseases (NCDs) such as diabetes (Tonstad et al., 2013), heart disease (Tong et al., 2019) and diverticular disease (Crowe et al., 2011), while following a low or no meat diet can help reduce the risk (Satija and Hu, 2018; Knuppel et al., 2019; Qian et al., 2019). This is particularly true of red and processed meat. In addition to being directly associated with increased risk of obesity (Rouhani et al., 2014), their association with an increased risk of colorectal cancer (Norat et al., 2005; Bradbury et al., 2020) contributed to the World Health Organization’s International Agency for Research on Cancer (IARC) 2015 classification of processed meat as carcinogenic to humans and red meat as “probably” carcinogenic (World Health Organisation, 2015). Some scientists disagree, however, suggesting that, “for most people, the health benefits are too small and uncertain to warrant reducing meat consumption” (Vernooij et al., 2021). Furthermore, other studies suggest that plant-based diets may also lead to certain health issues, with individuals following vegan diets more at risk of stroke (Tong et al., 2019) and fractures (Tong et al., 2020). As such, while some evidence suggests health benefits of limiting red and processed meat consumption, the health impacts of meat (whether in general or specific types) and plant-based consumption remain contested, with evidence inconclusive and researchers divided (Science Media Centre, 2019; Papier et al., 2021).

Although meat consumption levels remain high in the UK—in 2019 the average citizen consumed almost twice the amount of meat per capita than the average person around the world (OECD-FAO Agricultural Outlook, 2020)—consumption habits are changing: average daily UK meat consumption decreased by about 17% between 2008–09 and 2018–19 (Stewart et al., 2021). However, while consumers are eating less red meat, their intake of white meat has been increasing (OECD-FAO Agricultural Outlook, 2020; Stewart et al., 2021); this reflects other studies’ observations that in many regions chicken is widely being consumed at the expense of beef (Godfray et al., 2018). Key reasons for this changing behaviour include personal choices, driven by increasing public awareness about the potential health risks associated with red meat consumption and the negative environmental impact particularly of ruminant meat (Godfray et al., 2018). But economic stimuli also play a role, such as the higher price of red meat than poultry by weight in western countries (Lusk, 2016).

Socio-economic factors strongly influence people’s meat consumption levels. In many lower-income countries meat has traditionally been seen as an indicator of status and wealth (Happer and Wellesley, 2019). This relates to perceptions around Bennett’s law, where, as wealth and urbanisation increase, so too does the consumption of animal products (Bennett, 1941; Popkin, 1998). In contrast, in high-income western countries, lower meat consumption may increasingly be seen as an indicator of health-conscious lifestyles (Godfray et al., 2018).

2021 YouGov survey data on diet and news consumption in the UK reflect these international trends on a national scale (see Supplementary Material, Section 1). The survey found, first, that adults from lower-income (C2DE) communities were more likely to eat meat than those from higher-income (ABC1) groups (white respondents: ABC1 69%, C2DE 77%; BAME: ABC1 58%, C2DE 70%). Second, although around 25% of all participants had recently decreased their meat intake, this trend was greater among individuals from higher-income backgrounds (white: ABC1 24%, C2DE 22%; BAME: ABC1 28%, C2DE 25%). Given the association between red and processed meat intake and obesity (Rouhani et al., 2014), the higher levels of meat consumption among lower-income groups may relate to Holmes (2021)’s findings that in 2019/20 rates of obesity-related hospital admissions were 2.4 times greater in the most deprived areas of England than in the least deprived.

While the YouGov data suggest that mainstream media play a relatively minor role in influencing consumers’ dietary decisions when compared to family and friends (see Supplementary Material, Section 1), a large amount of scholarship suggests that the media exerts a major influence in setting people’s views about which issues are important and shaping the way these are discussed, both in general (Xinsheng et al., 2009) and around dietary and environmental issues in particular (Happer and Wellesley, 2019). For this reason, an awareness of how meat and plant-based foods are portrayed from a nutritional perspective in mass media particularly (though not exclusively) consumed by lower-income groups can provide us with a greater understanding of popular narratives and debates that exist around the health impacts of meat or plant-based consumption.

### Previous research

Despite the role that health plays in shaping an individual’s dietary habits (Mullee et al., 2017; Jones, 2020), few studies have examined media coverage of the impact of meat on health. In Ihekweazu (2021)’s study on depictions of various dietary items in New York Times articles between 1996 and 2016, although meat played only a minor role, narratives around meat-free diets were found to be exclusively positive, while those around red meat and processed meat were found to be predominantly and exclusively negative, respectively. Focusing on the meat-health nexus, Leroy et al. (2018) examined 1,310 MailOnline articles published between 2001 and 2015. They found that 52% reported negative associations between meat and health, 35% reported positive associations, while 13% mentioned both positive and negative aspects. Although these findings suggest a dominant anti-meat sentiment, they observed that meat was presented slightly more positively than negatively between 2001 and 2003—likely due to the policy of reassurance following the bovine spongiform encephalopathy (BSE) crisis—and that the heterogeneity of voices throughout their corpus added to the “rowdy and dissonant” nature of the meat-health debate. This supports other research which has shown meat consumption to be a highly contested subject in social media, for example in the popularity of “#yes2meat” tweets around the launch of the EAT-Lancet report (Garcia et al., 2019). Nevertheless, the evidence for the mainstream media’s tendency towards anti-meat sentiment from a health perspective lends credence to Morris (2018)’s observation that “a shift is taking place in the status of meat within UK society” with print media “working in support of de-meatification”.

We aim to build on this area of research by considering more contemporary media depictions of the health impacts of meat across a wide variety of online news consumed by all demographics in the UK, but particularly popular among lower-income groups—the group most likely to eat meat and with poorer health outcomes (Choi et al., 2020). In addition to determining the level of coverage of the meat-health nexus in various media, this study aims to uncover representations of meat by considering (1) health-based narratives for/against meat-eating, (2) dietary solutions or recommendations for optimal health, and (3) whether articles are more in favour of or against eating meat. We use these quantitative results, first, to determine whether there is any correlation between type of news site (e.g. left- versus right-wing) and article sentiment, and second, to uncover key themes present in our dataset to shape our qualitative analysis.

## METHODS

### Data sources

Using 2020 YouGov survey data on digital news consumption (Newman, 2020), we selected the top five news sites accessed by the C2DE (lower-income) demographic in the UK in the week leading up to the survey—BBC, 35%; Guardian, 12%; MailOnline, 11%; Sky News, 9%; Sun Online, 8%—and three that followed at 5%: Mirror, BuzzFeed and LAD Bible. Our sample shows a broad range of media types and political leanings: two left-wing (Mirror and the Guardian) and two right-wing newspapers (MailOnline and Sun) (Smith, 2017), two impartial broadcasters (BBC and Sky) (Ofcom, 2021), and two newer, niche sites that have not been studied widely in media research (BuzzFeed and LAD Bible).

### Data collection

The search term “meat” was used to collect articles from 2019. This provides a 1-year snapshot of narratives at a time of increasing popular interest in veganism (The Vegan Society, 2021). A number of key reports were also published during this year—such as the EAT-Lancet report (Willett et al., 2019) and several UK Biobank studies (Bradbury et al., 2020; Knuppel et al., 2020)—which are known to drive up media coverage (Kristiansen et al., 2021).

For the online newspapers (Guardian, MailOnline, Mirror, Sun) the online database Factiva was used to gather articles. For the online news outlets (BBC, Sky, LAD Bible, BuzzFeed), articles were retrieved from Google News using the “site:” operator, which displays results from the indexed pages from the specified website (for example, “meat site:bbc.co.uk” retrieves meat-related articles from the BBC website). Data collection was undertaken by both researchers over several days. Minor fluctuations occurred in the daily total number of search results (due, for example, to duplicate articles in Factiva, and to Google algorithms and user personalisation) and for this reason the total number of articles retrieved was not recorded.

The two researchers divided the news outlets between them and agreed on inclusion and exclusion criteria to ensure only relevant meat-related articles were collected. Only news, opinion pieces and features articles were retained; blogs and letters to the editor were excluded. Articles had to refer to meat (whether in general or to specific types, e.g. beef) or plant-based alternatives in four or more lines to ensure article salience. 947 meat-related articles were thus identified; they formed the dataset of a broader research project examining representations of meat in the media (see Mroz and Painter (forthcoming) for portrayals of the impact of meat on the environment from this data).

### Data analysis

Data analysis software NVivo was used to categorise articles into topics according to salient themes in the opening five lines; articles could belong to more than one topic. Full topic distribution is detailed in Mroz and Painter (forthcoming).

128 articles (13.5%) were identified as belonging to the health and nutrition topic. They were distributed thus: MailOnline, *n* = 45; Sun, *n* = 24; Mirror, *n* = 17; BBC, *n* = 15; Guardian, *n* = 13; Sky News, *n* = 7; LAD Bible, *n* = 5; BuzzFeed, *n* = 2.

A codebook (see Supplementary Material, Section 2) was developed to facilitate detailed content analysis for each of these articles. It contained 42 variables divided into three main areas of inquiry:

i. Anti-meat and pro-meat narratives;
ii. Dietary solutions, recommendations and/or advice;
iii. Overall article sentiment: (a) anti-meat (if anti-meat or pro-plant-based narratives were dominant); (b) pro-meat (if pro-meat or anti-plant-based narratives were dominant); and (c) balanced or neutral (if they featured a similar number of pro-/anti-meat narratives, contained no stance or an absence of narratives).

The two researchers coded articles according to the variables in the codebook. The Cohen’s Kappa inter-coder reliability score (considered to be a more robust method than simple percent agreement calculation) was >0.8 (strong agreement) for all but seven variables. Four of these scored between 0.7 and 0.8 (substantial agreement); the remaining three scored between 0.48 and 0.64, due only to one discrepancy between the coders in a dominant sequence of zero coding (a result often given by using Cohen’s Kappa): a simple percentage calculation gave >90% agreement. For all variables which scored <0.8, the coders discussed the discrepancies to achieve agreed coding.

The authors used the results of the quantitative method to guide an additional layer of qualitative analysis. In common with general qualitative approaches (Metag, 2016), this involved a more interpretative and contextualised reading of a small sample of the material, to give nuanced insights into the media’s representation of the meat-health nexus.

### Researcher perspective

The two researchers have extensive experience in media analysis of health and environmental topics, and have published widely in peer-reviewed journals in these areas.

## RESULTS

### Pro- and anti-meat narratives

**Fig. 1:**
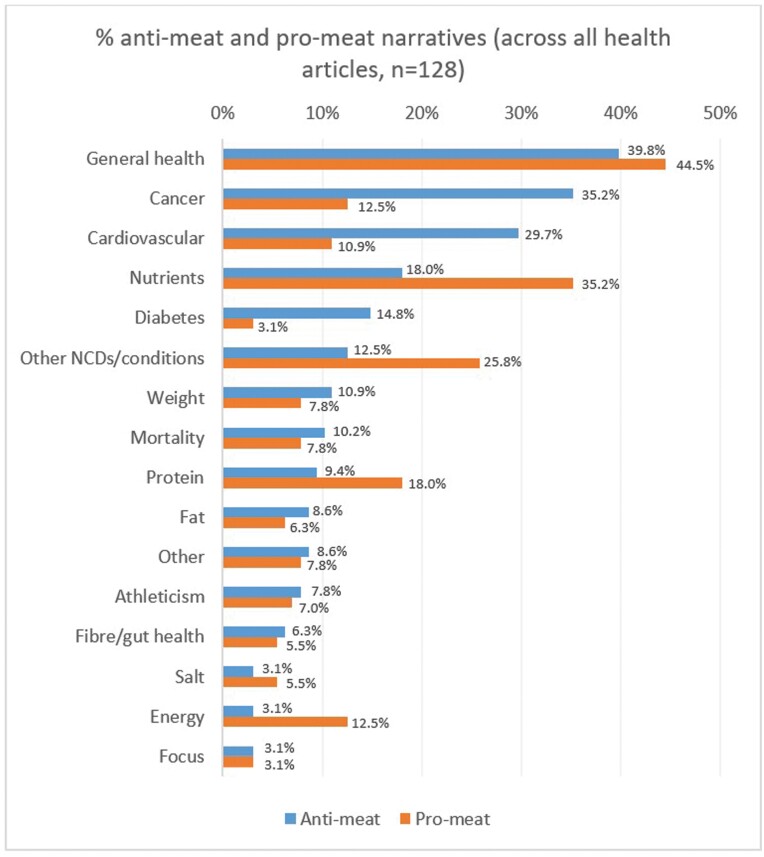
Percentage distribution of anti-meat and pro-meat narratives across all health articles.

Figure 1 shows the percentage distribution of pro- and anti-meat narratives across all health and nutrition articles. The most common anti-meat narrative concerned general health: that meat (including specific types of meat, usually red) is unhealthy, or that plant-based diets are healthy (present in 39.8% of articles). This was followed by the narratives that meat increases the risk of cancer (35.2%) and cardiovascular issues (29.7%).

The dominant pro-meat narrative argued generally that meat is healthy, or that plant-based diets are unhealthy or not healthier than meat (44.5%). This was followed by the arguments that meat contains important nutrients which plant-based diets often lack (35.2%) and that meat can help to improve or relieve (or that plant-based diets can increase the risk of) NCDs or other health issues, such as kidney failure, hormonal imbalances and autoimmune conditions (25.8%).

The distribution of pro- and anti-meat narratives is largely similar across news sites (see Supplementary Material, Section 3, for the top three pro-/anti-meat narratives by outlet). General health, cancer and cardiovascular issues were the three most common anti-meat narratives in six outlets: the BBC, Guardian, MailOnline, Sun, Mirror and Sky. The increased risk of diabetes from meat consumption and the adequacy of plant-based diets for nutrient intake were the other common narratives in these outlets. General health was also prominent in LAD Bible, and cardiovascular issues in BuzzFeed (but its sample size was very low).

General health featured among the three most common pro-meat narratives in seven news sites (BuzzFeed being the exception). The inadequate nutrition of plant-based diets featured in the top three pro-meat narratives in all outlets, and other NCDs or health issues in all but one (BBC).

### Solutions, recommendations, advice

**Fig. 2:**
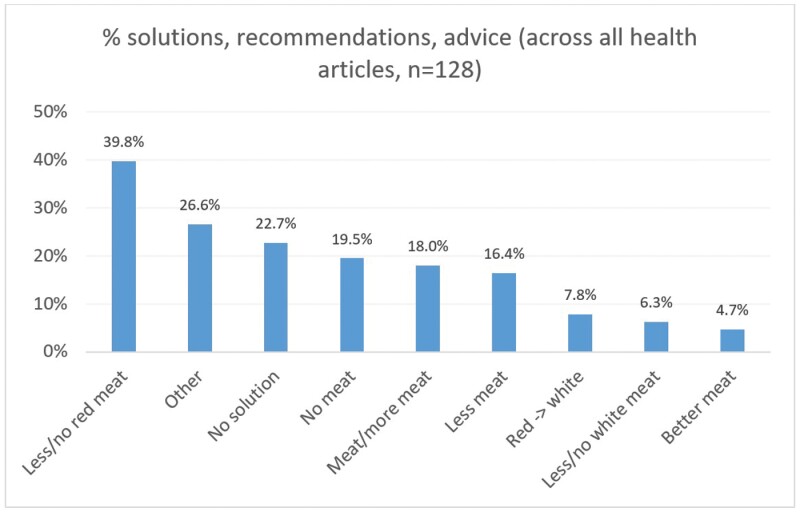
Percentage distribution of solutions, recommendations and advice across all health articles.

The distribution of solutions, recommendations and advice across all health and nutrition articles can be seen in Figure 2. The most common solution was to “eat less/no red meat” (39.8%). This was followed by “other solutions”, such as to avoid processed meat, strive for a balanced diet, or for the government to introduce a meat tax (26.6%). Just under a quarter of articles contained no solution (22.7%). “Eat no meat” (19.5%) and “eat (more) meat” were other common suggestions (18.0%). Only 4.7% of articles recommended switching to better (lean or unprocessed) meat, with 3.1% advising this together with a reduction in meat in general and/or red meat in particular.

Solutions were largely consistent across media outlets (see Supplementary Material, Section 4). “Eat less/no red meat” was the most common solution or recommendation for the Guardian, MailOnline, Mirror and Sun, and was the joint most common one for Sky.

### Sentiment

**Fig. 3:**
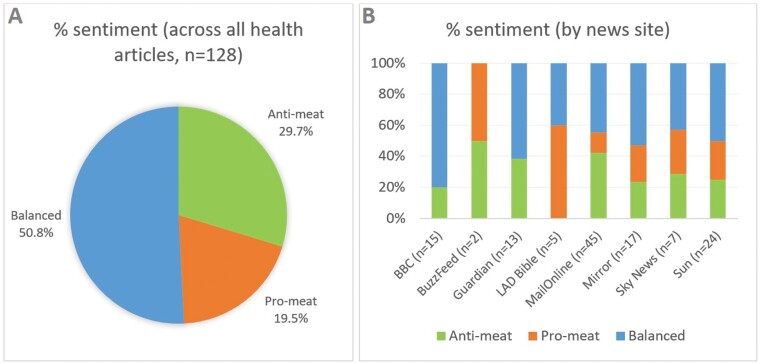
**(a)**: Percentage distribution of meat consumption sentiment across all health articles. **(b)**: Percentage distribution of meat consumption sentiment by news site.

Just over half the articles contained a balanced or neutral sentiment (50.8%). 29.7% were anti-meat, while 19.5% were pro-meat (see Figure 3a).

Sentiment distribution by news site can be seen in Figure 3b. Four news outlets were neutral or balanced in their reporting. BuzzFeed had an equal proportion of anti-meat and pro-meat articles (50%), with none neutral or balanced (but again, its sample size was very low). The Mirror, Sky News and the Sun were also balanced in their reporting, with all having the same percentage of anti-meat articles as pro-meat (23.5%, 28.6% and 25.0%, respectively), and the rest of their articles neutral or balanced.

Three news outlets were slightly anti-meat. MailOnline (42.2%) and the Guardian (38.5%) had the highest proportion of anti-meat articles, after BuzzFeed; unlike BuzzFeed, however, the Guardian featured no pro-meat articles. BBC also had no pro-meat articles; it had the greatest proportion of neutral or balanced articles (80.0%), with its remaining articles (20.0%) anti-meat. Although MailOnline had some pro-meat articles, it had the lowest proportion (13.3%) after Guardian and BBC.

LAD Bible had the highest percentage of pro-meat articles (60.0%), and was the only media outlet not to have any anti-meat articles.

### Qualitative results

The results of our quantitative method guided our qualitative analysis by revealing three recurring themes, analysed below as case studies. The first was the correlation between red meat and cancer, driven both by dominance of the narrative that meat can increase the risk of (colorectal) cancer and the dominant solution, to “eat less/no red meat”. The second was plant-based options, chosen due to polarised narratives and the similar, though slightly greater, proportion of articles that displayed anti-meat sentiment over pro-meat. For both case studies we focus on two studies that dominated coverage to consider the media’s reporting of scientific risk and uncertainty. Other articles focused on personal narratives rather than scientific research, which led to our third case study, individual dietary choice: this was prominent in articles containing polarised solutions, “eat no meat” and “eat (more) meat”, present in similar proportions.

#### Red meat and cancer

Two major studies examining the impact of red meat on health released in 2019 were reported on in the media: the UK Biobank study (Bradbury et al., 2020) and the Johnston et al. paper (Johnston et al., 2019).

The Biobank study suggested a strong correlation between red meat consumption and increased risk of colorectal cancer. Its conclusion, that “[c]onsumption of red and processed meat at an average level of 76 g/day that meets the current UK government recommendation (90 g/day) was associated with an increased risk of colorectal cancer” (Bradbury et al., 2020), appeared widely. Articles, such as “Two rashers of bacon a day raises bowel cancer risk by a fifth” (Sky News, 2019b), commonly pointed to a 20% increase in risk without further explanation. A blogpost in the BMJ (Shaw, 2019) criticised the “misleading misreporting of statistics” in the media’s reporting of the study. It drew attention to the fact that no media coverage explained that 20% was for relative risk increase, not absolute risk increase, before clarifying that “in individual terms the absolute risk increase is 0.08%, not 20%”. It also criticised the researchers for not better clarifying these findings. However, although the absolute risk increase for an individual might be considered small, the impact of small increases in individual risk adds up to a large population impact, particularly for a condition like colorectal cancer (Science Media Centre, 2019), a point that was also absent from the coverage.

Various voices supported the study, including Dr Julie Sharp from Cancer Research UK (Sky News, 2019b), while others, like Dr Carrie Ruxton, a dietician and member of the industry-funded Meat Advisory Panel, defended meat consumption. Dr Ruxton suggested—correctly—that “a range of lifestyle factors have a significant impact on the risk of bowel cancer” (BBC, 2019). The study did find that alcohol consumption can increase the risk while higher fibre intake from wholegrains can minimise it (Bradbury et al., 2020), but few articles drew attention to these findings.

In contrast, Johnston et al. (2019) recommended that adults continue their current intake of processed and unprocessed red meat. This study faced criticism (The Nutrition Source (Harvard TH Chan School of Public Health), 2019) which made its way into the media coverage. In one article Professor of Epidemiology Tim Key criticised the report for contradicting general scientific consensus, explaining that its authors “found the same evidence of an effect but they think it is so modest that it isn’t worth recommending we do anything about it” (Sky News, 2019a). To question the study’s findings further, the same article drew attention to the aforementioned UK Biobank study (Bradbury et al., 2020) and the idea that “just one rasher of bacon per day” could “increase the risk of bowel cancer by 20%” (Sky News, 2019a). Other articles drew attention to the Department of Health’s advice that consumers should eat “no more than 70 g a day” and Public Health England’s recommendation that consumers “eat less” red meat (Gallagher, 2019).

Despite the criticism, some voices were quick to rejoice in the study’s findings. Television presenter Piers Morgan, well-known for his anti-vegan stance, was quoted in the Sun:

Do you know what it says? Carry on eating meat, it says. In fact, if you don’t eat meat, that could be more harmful in the long-term. […] Vegetarians, eat your gruel. Go ahead and do it. But it no longer gives you health benefits (to cut out meat) (Gallagher, 2019).

Although we did not code for the specific sources quoted in each article, we found that the majority of researchers and health professionals (with the exception of Dr Ruxton and the authors of the Johnston et al. study) recommended a reduction in red meat consumption, with non-health-related individuals (such as a controversial television presenter) arguing against a reduction.

#### Plant-based options

The reporting of the EPIC-Oxford study (Tong et al., 2019) and the EAT-Lancet report (Willett et al., 2019) about plant-based eating shows that the topic is as contested in our media sample as eating red meat.

The EPIC-Oxford study found that “fish eaters and vegetarians had lower rates of ischaemic heart disease than meat eaters, although vegetarians had higher rates of haemorrhagic and total stroke” (Tong et al., 2019). However, given the generally accepted associations between higher proportions of plant-based foods and improved heart health outcomes (Bergeron et al., 2019), media articles tended to focus on the other major finding from this study: that plant-based diets can increase the risk of stroke. One example, “Vegans and vegetarians may have higher stroke risk”, despite the anti-plant-based sentiment of the title, discussed the benefits of plant-based diets for heart disease (Parkinson, 2019). Although the article correctly highlighted the associative nature of the study, which “cannot prove whether the effect is down to their diet or some other aspect of their lifestyle”, discussions of uncertainty were largely overshadowed (and, indeed, supported) by general nutritional guidance, for example advising plant-based eaters to be mindful of their nutrient intake.

The EAT-Lancet report recommended a significant reduction in red meat and poultry intake among western consumers. Like the EPIC-Oxford study, the EAT-Lancet report contained some uncertainty. However, it suggested that by “applying a precautionary and risk perspective”, its recommendations could be placed “at the lower end of the scientific uncertainty range” (Willett et al., 2019). Some scientific commentators have criticised the report for not “fully account[ing] for statistical uncertainty” (Zagmutt et al., 2020). However, only one article in our sample loosely raised the issue of uncertainty in the report’s “one-size-fits-all” dietary guidance, but otherwise regarded it as a “useful piece of work” (Anthony, 2019).

While some academic literature contested the EAT-Lancet’s findings from a science-based perspective (Zagmutt et al., 2019, 2020), right-leaning media articles contested its recommendations from a libertarian one. One article in MailOnline talked of “bacon lovers [reacting] in horror to new guidelines” (Blott and Spencer, 2019). Although it highlighted the report’s findings that “the adoption of a ‘planetary health diet’ is vital to feed the world’s booming population without destroying the environment”, some consumers claimed that “life wouldn’t be worth living” on a half rasher of bacon per day. A similar article in the Sun with the headline “Bacon Batty” described the EAT-Lancet as a “‘nuts’ nanny state report” (McDermott, 2019).

#### Individual dietary choices

Where the aforementioned articles report on scientific studies, solutions like “eat less/no red meat” by healthcare professionals are offered more as general advice than dogma. In contrast, other articles feature anecdotes in which individuals offer (often dogmatic) advice from personal, not professional, experience. There is far more polarisation in their accounts, being either pro-vegan or pro-carnivore.

These extreme narratives are largely gendered, with the pro-vegan narratives usually coming from young, previously meat-eating males, and the pro-carnivore narratives from young, previously vegan females. The narratives differ accordingly. The male narratives tend to focus on possible improved sexual and athletic performance on plant-based diets. One bodybuilder and gym owner discusses his excitement “for the opportunity to go to his next bodybuilding and power-lifting competitions to show off his vegan body” (McCleery, 2019). The female narratives tend to focus on switching from vegan to carnivore diets to improve autoimmune conditions. One woman found that within a week of adopting a carnivore diet, she “felt amazing and for the first time in a long time my body was free of pain” (Ball and Lavender, 2019).

While these two articles rely solely on an individual’s account, most articles of this type include dietary counterarguments from health and nutrition practitioners. A leading UK eye surgeon, in response to a bodybuilder who claims a vegan diet improved his eyesight, suggests he have “his eyes examined again to rule-out anything untoward” (Wood, 2019). A nutritionist, in response to the increasing popularity of carnivore diets, particularly raw ones, explains that a “raw meat diet is very restricted and additionally increases the risk of nutritional deficiencies” (Jolly, 2019). As such, while a particular restrictive diet might work for an individual, they are not readily recommended by health professionals for all.

## DISCUSSION

Our findings contribute to, and in some cases challenge, a nascent area of scholarship. Like in Leroy et al. (2018)’s study of MailOnline articles, we find a multiplicity of anti- and pro-meat narratives, even on the same pathologies (e.g. cancer). Our dominant solution, to “eat less/no red meat” (40% of articles), aligns with the negative health perceptions of red and processed meat observed by Ihekweazu (2021) in New York Times articles. However, we observe contestation, rather than “exclusively positive” narratives, around plant-based diets, as seen in the similar proportion of articles containing recommendations to “eat no meat” and “eat (more) meat” (19.5% versus 18%). This contestation is perhaps best illustrated by the presence of two opposing narratives—“soy is poison” and “soy is the best health food ever”—in the same article (Edmonds, 2019), inferring a modern-day, vegan equivalent of “one man’s meat is another’s poison”.

Like Leroy et al. (2018), who found a greater proportion of MailOnline articles reporting on the negative health impacts of meat than on the positive (52% versus 35%), we observe a slightly greater proportion of all articles containing anti-meat sentiment than pro-meat (30% versus 20%). However, whereas only 13% of their articles contained balanced sentiment, just over half of ours were balanced or neutral (45% in MailOnline). Although this may be partly due to our retention of neutral articles (containing no anti-/pro-meat health narratives) and different coding application, the lower proportion of articles containing anti-/pro-meat sentiment, and higher proportion containing balanced/neutral reporting, may reflect a greater plurality of views, and less polarisation, across a broader range of media outlets.

The media reporting in our sample generally reflects, and accurately reports on, the scientific research. However, our qualitative analysis of three themes uncovers several issues that should be addressed to help improve public understanding around diet and nutrition. First, many articles that report on scientific studies and/or dietary choices fail to contextualise these “against the background of the broader scientific literature and established facts” (Smith et al., 2016). It is important for the reader to be aware if the results of a new study align with existing literature, or if they are an outlier and should thus be approached with caution (Ihekweazu, 2021). Second, issues often arise in the media’s reporting of scientific results. We see this in the reporting of absolute versus relative risk, as in the case of the increased risk of colorectal cancer, and of statistical uncertainty, as seen in the lack of discussion on this topic in articles reporting on the EPIC-Oxford study or EAT-Lancet report. Third, articles featuring personal stories often fail to show whether individual dietary decisions are supported by scientific research. The inclusion of scientific evidence and/or advice from accredited professionals would be beneficial particularly for individuals considering adopting more extreme diets, such as carnivore or raw vegan, both of which lack certain important nutrients. Finally, the cherry-picking of research papers and anecdotal evidence to suit editorial approaches persisted in LAD Bible, targeting the young male reader. One article (Shepherd, 2019) reported the results of a scientific study, published five years prior, which found that “a vegetarian diet is associated with poorer health” (Burkert et al., 2014), to support this pro-meat stance.

Since many non-specialist journalists often do not have the same technical knowledge or awareness of the research landscape as academic researchers, it is important for both parties to work both independently and together for correct information to be communicated accurately to the public. There are several ways these obstacles can be overcome and reliable communication achieved. First, improved dialogue between journalists and the researchers involved in a study would facilitate improved contextualisation of results against the wider academic background; dialogue with researchers unaffiliated with the study would provide an objective assessment of the research in question (Ihekweazu, 2021). The London-based Science Media Centre facilitates such dialogue and provides guidelines that support accurate reporting by non-specialist journalists (Science Media Centre, n.d.). Second, researchers should ask to see and edit press releases about their studies to improve accuracy and clarity of their results (Schwitzer et al., 2005). Using the researcher’s wording would be particularly useful in discussions of risk and uncertainty as it would leave less room for misinterpretation (Shaw, 2019). Third, while it is to be expected that certain news outlets publish articles based on personal narratives, commentaries by trusted healthcare professionals and/or scientists, supported by academic literature and foregrounded in the articles, would enable consumers to make informed dietary choices based on scientific rather than anecdotal evidence. Finally, the cherry-picking of anecdotal stories and scientific research should be avoided. While we appreciate that suggesting that a media outlet should soften or even change its editorial approach would be an ambitious recommendation, we encourage unbiased reporting on health-related concerns through the reporting of recent, trusted scientific studies, supported by dialogue with researchers and health practitioners.

Multiple factors shape journalistic output, including macro-level pressures coming from advertisers and media owners, meso-level organisational demands (pressures to be brief, to produce stories quickly on different platforms, and to gain readers and clicks) and, at the micro-level, the individual journalist’s prejudices and beliefs (Shoemaker and Reese, 1995). However, attention to these recommendations and others would help to minimise misleading information about the risks and benefits of meat-eating. Although numerous factors contribute to the close relationship between obesity and deprivation (Holmes, 2021), reporting on scientific, nutrition-based findings accurately, comprehensively and critically can help to reduce potentially damaging perceptions around diet and help to improve health outcomes among all sectors of UK society, including those on lower incomes.

### Strengths, limitations, and future research

Previous research on media representations of the meat-health nexus is scarce and limited to analysis of individual news outlets. A key advantage of the present study is our consideration of a broad range of online news sites, which enables us to consider how attitudes towards the relationship between meat and health differ depending on a media outlet’s editorial approach. Analysis of news articles from 2019 offers a more contemporary snapshot of British media attitudes towards the subject, as well as insights into coverage of various nutrition-related reports (e.g. the EAT-Lancet), at a time of increasing interest in plant-based diets.

Though useful data collection tools, Factiva and the Google “site:” operator do not always retrieve all articles pertinent to a search. The coders used an iterative process to agree on coding and ensured a high inter-coder reliability score was met; however, some discrepancies might still have occurred given the large number of articles and variables. For scoping purposes, the present study was limited to UK online news sites. Future studies can build on this research by considering alternative media (e.g. social media) and different geographical and health contexts (e.g. the COVID-19 pandemic).

## CONCLUSION

This study examined representations of meat from a health perspective in eight of the most widely consumed online news sites by lower-income groups in the UK. With the exception of some extreme pro-vegan and pro-carnivore narratives in the tabloid and niche media, we note little polarisation across the British news landscape, with most news outlets either neutral or slightly anti-meat in their coverage of the meat-health nexus. Significantly, we find that political leaning is not closely correlated with sentiment: for example, the left-wing Guardian, the impartial BBC, and the right-wing MailOnline, were all slightly anti-meat. But ideology and target audience appear to play a role in LAD Bible: aimed at a largely young, male audience, it was the only pro-meat outlet from our sample. This suggests that meat continues to play a role in shaping perceptions of masculinity in UK society, as it does in other Anglophone countries (Rothgerber, 2013; Carroll et al., 2019; Mesler et al., 2022). Improved reporting by journalists on the relationship between meat consumption and health can help to improve health outcomes in the UK, including among lower-income groups.
